# Hyaluronic Acid Supplement as a Chondrogenic Adjuvant in Promoting the Therapeutic Efficacy of Stem Cell Therapy in Cartilage Healing

**DOI:** 10.3390/pharmaceutics13030432

**Published:** 2021-03-23

**Authors:** Chin-Chean Wong, Shi-Da Sheu, Pei-Chun Chung, Yi-Yen Yeh, Chih-Hwa Chen, Yen-Wei Chang, Tzong-Fu Kuo

**Affiliations:** 1 Department of Orthopedics, Shuang Ho Hospital, Taipei Medical University, New Taipei City 23561, Taiwan; b8701153@tmu.edu.tw (C.-C.W.); 18028@s.tmu.edu.tw (C.-H.C.); 2 Department of Orthopedics, School of Medicine, College of Medicine, Taipei Medical University, Taipei 11031, Taiwan; 3 Research Center of Biomedical Devices, Taipei Medical University, Taipei 11031, Taiwan; 4 International Ph.D. Program for Cell Therapy and Regenerative Medicine, College of Medicine, Taipei Medical University, Taipei 11031, Taiwan; 5 Non-Invasive Cancer Therapy Research Institute of Taiwan, Taipei 10489, Taiwan; 6 Department of Chinese Medicine, E-Da Cancer Hospital, Kaohsiung 82405, Taiwan; ed112824@edah.org.tw; 7 School of Veterinary Medicine, National Taiwan University, Taipei 10617, Taiwan; r02629012@ntu.edu.tw; 8 School of Biomedical Engineering, College of Biomedical Engineering, Taipei Medical University, Taipei 11031, Taiwan; yan0228@tmu.edu.tw; 9 School of Medicine, College of Medicine, Taipei Medical University, Taipei 11031, Taiwan; 10 Office of Physical Education, Asia University, Taichung 41354, Taiwan; david@asia.edu.tw; 11 Department of Post-Baccalaureate Veterinary Medicine, Asia University, Taichung 41354, Taiwan

**Keywords:** bone marrow stem cells, cartilage, hyaluronic acid, injection, repair

## Abstract

The main aim of this study is to investigate the therapeutic efficacy of direct intra-articular injection of bone-marrow-derived stem/stromal cells (BMSCs) and the adjuvant role of hyaluronic acid (HA) in facilitating rabbit articular cartilage repair. First, rabbit BMSCs were treated with a medium containing different concentrations of HA. Later, HA’s influence on BMSCs’ CD44 expression, cell viability, extracellular glycosaminoglycan (GAG) synthesis, and chondrogenic gene expression was evaluated during seven-day cultivation. For the in vivo experiment, 24 rabbits were used for animal experiments and 6 rabbits were randomly allocated to each group. Briefly, chondral defects were created at the medial femoral condyle; group 1 was left untreated, group 2 was injected with HA, group 3 was transplanted with 3 × 10^6^ BMSCs, and group 4 was transplanted with 3 × 10^6^ BMSCs suspended in HA. Twelve weeks post-treatment, the repair outcome in each group was assessed and compared both macroscopically and microscopically. Results showed that HA treatment can promote cellular CD44 expression. However, the proliferation rate of BMSCs was downregulated when treated with 1 mg/mL (3.26 ± 0.03, *p* = 0.0002) and 2 mg/mL (2.61 ± 0.04, *p* = 0.0001) of HA compared to the control group (3.49 ± 0.05). In contrast, 2 mg/mL (2.86 ± 0.3) of HA treatment successfully promoted normalized GAG expression compared to the control group (1.88 ± 0.06) (*p* = 0.0009). The type II collagen gene expression of cultured BMSCs was significantly higher in BMSCs treated with 2 mg/mL of HA (*p* = 0.0077). In the in vivo experiment, chondral defects treated with combined BMSC and HA injection demonstrated better healing outcomes than BMSC or HA treatment alone in terms of gross grading and histological scores. In conclusion, this study helps delineate the role of HA as a chondrogenic adjuvant in augmenting the effectiveness of stem-cell-based injection therapy for in vivo cartilage repair. From a translational perspective, the combination of HA and BMSCs is a convenient, ready-to-use, and effective formulation that can improve the therapeutic efficacy of stem-cell-based therapies.

## 1. Introduction 

Cartilage injuries are a common and important source of knee dysfunction and present major clinical challenges to both clinicians and patients. Since articular cartilage is an avascular tissue, its self-healing capacity is limited [1]. As a consequence, cartilage defects are prone to progress and inevitably turn into catastrophic osteoarthritis at a later stage [2,3]. For severe joint destruction, cartilage can be replaced by an artificial endoprosthesis, namely joint arthroplasty. In contrast, for patients with mild-to-moderate joint destruction, searching ways to slow down further deterioration of the damaged articular surface is important [4]. In fact, many therapeutic strategies have been developed to enhance cartilage healing by introducing cells or tissues. These includes microfracture, osteochondral autograft transplantation, and autologous chondrocyte implantation [5,6]. However, surgical outcomes are still controversial, and thus, effective treatment is still lacking [7,8,9]. 

Recently, cell-based cartilage regeneration strategies using chondrocytes have presented a feasible and promising alternative, but they require harvesting articular cartilage specimens from the non-weight-bearing zone of the joint [9]. Nevertheless, the amount of healthy cartilage available for biopsy is limited [2,3]. Thus, a large number of chondrocytes are needed to generate a functional biological construct to implant into the defect area. In vitro expansion has been reported to result in chondrocyte dedifferentiation, resulting in fibrous and mechanically inferior fibrocartilage formation [10,11]. In recent years, mesenchymal stem cell therapy has been used to treat cartilage injury, and the results are encouraging [12]. Bone-marrow-derived stem/stromal cells (BMSCs) hold great potential as a promising candidate to treat cartilage defects, not only because of their multiple differentiation capacity, but also because of their ability to home into injured tissues in response to signaling pathways [13,14,15]. Moreover, stem cells can be isolated from various sources, like bone marrow and adipose tissue, with minimal donor site morbidity and in greater numbers than chondrocytes. Additionally, under appropriate culture conditions, stem cells can be expanded in vitro while maintaining their chondrogenic potential [16]. Several studies have demonstrated the efficacy of intra-articular (IA) injection of cultured BMSCs in promoting healing of chondral defects, not only in preclinical animal studies, but also in pilot human clinical trials [17,18,19,20,21]. 

Nevertheless, the success of stem-cell-based therapy for cartilage repair or regeneration relies on not only obtaining an appropriate cell source but also identifying a strategy to maintain and localize the cells at the lesion site. Hyaluronic acid (HA) is one of the main components of the extracellular matrix (ECM) of cartilage and is present in a high concentration in the synovial fluid of joints [22,23]. For decades, HA has been widely used as a viscosupplement in treating osteoarthritic joints by providing joint lubrication to alleviate pain by reducing the friction of the joint and improving the viscoelasticity of the synovial fluid [22,23]. HA also plays a significant role in a multitude of biological processes involving cell migration, proliferation, differentiation, and wound healing [23]. Moreover, the major characteristics of HA include its high molecular weight (from 1 kDa up to 9 MDa) and hydrophilic nature, rendering it a suitable cell carrier when combined HA and stem cells are concurrently delivered into a joint through intra-articular (IA) injection [24]. 

Although the clinical application of stem-cell-based therapies for treating cartilage defects is promising, some controversies and inconsistences remain that may be attributed to different culture methods, the way of cell delivery, and different combined formulations. To increase the applicability of cell-based injection therapy, we aim to evaluate the therapeutic efficacy of HA alone, BMSCs alone, and combined HA and BMSCs in promoting the healing of cartilage defects in a rabbit model. Moreover, we intend to delineate the individual therapeutic role of BMSCs, HA, or their combination in this stem-cell-based therapy regimen. In the present study, we hypothesize that HA can serve as an effective chondrogenic adjuvant by virtue its chondroprotective and cytoprotective effects. We speculate that combined HA and BMSCs would be more effective than BMSCs alone in promoting the healing of chondral defects after in vivo transplantation. 

## 2. Materials and Methods 

### 2.1. Study Design and Ethics Statement

All procedures of bone marrow stem cell isolation and surgery on experimental animals were carried out according to the Guide for the Care and Use of Laboratory Animals and was approved by the Institutional Animal Care and Use Committee (IACUC approval: NTU-102-EL-92).

### 2.2. Harvest and Cultivation of Bone Marrow Stem Cells

Bone marrow aspirates were obtained aseptically from femurs of five skeletally mature female rabbits (age 14–16 weeks; weight 3–3.5 kg). Briefly, bone marrow specimens were collected from the disposed aspirates using a 10 mL syringe. The aspirates were immediately mixed with 0.5 mL of sodium-heparin (10,000 U/mL) and diluted in equal volumes of PBS. The cell suspension was then fractionated on Lymphoprep (Fisher Scientific, Goteborg, Sweden) and centrifuged at 400× *g* for 30 min. The interface fraction enriched with BMSCs was collected and plated onto a 10 cm dish containing 10 mL of α-Modified Eagle’s Medium (αMEM) containing 10% of fetal bovine serum (FBS) (Gibco, Paisley, UK) and 1X P/S/A (penicillin/ streptomycin/fungizone). After washing out non-adherent hematopoietic cells, the adherent BMSCs were cultured in 5% CO_2_ at 37 °C with the medium changed every 3–4 days. When the cells reached 80% confluence, they were trypsinized and passaged into new 10 cm dishes at a cell density of 5 × 10^5^ cells/dish. The cells were sub-cultured till passage 2 (P2). 

### 2.3. Flow Cytometry Analysis 

BMSCs were fixed with ethanol overnight at –20 °C. Aliquots of 5 × 10^5^ cells were incubated with each of the fluorochrome-conjugated antibodies against a panel of cell surface markers, including CD31-FITC (AB9498, Abcam, Cambridge, MA, USA), CD45-FITC (MCA808GA, Bio-Rad, Hercules, CA, USA), CD44-FITC (AB 119335, Abcam, USA), CD73-FITC (AB 175396, Abcam, USA), and CD90-FITC (BD 554895, BD Biosciences, San Jose, CA, USA) at 4 °C. Cells were resuspended in Con’s tube (BD) containing 200 µL of PBS/1% bovine serum albumin (BSA; A11133, Invitrogen, Carlsbad, CA, USA). Then, the cells were washed and stained with R-phycoerythrin (PE)-conjugated goat anti-mouse Immunoglobulin (Ig) (550589, BD), Alexa-Fluor-647-conjugated goat anti-rat IgG (ab150159, Abcam), and DyLight-488-conjugated donkey anti-rabbit IgG (SA5-10038, Thermo, Waltham, MA, USA) secondary antibodies at 4 °C for 30 min and analyzed by flow cytometry using the FACScan system (FACSAria, Becton Dickinson, Franklin Lakes, NJ, USA). 

### 2.4. Differentiation Assay 

The differentiation potential of BMSCs toward osteogenic, chondrogenic, and adipogenic lineages was assessed. P2 BMSCs treated with standard culture medium served as controls. For osteogenic differentiation of BMSCs, cells were cultured with an osteogenic medium containing 10% FBS, 50 µg/mL of L-ascorbate-2-phophate (A8960, Sigma-Aldrich, St. Louis, MO, USA), 10^−7^ M dexamethasone (D4902, Sigma-Aldrich), and 10 mM β-glycerophosphate (G9422, Sigma-Aldrich). After culturing for 3 weeks, cells were washed twice with PBS and fixed with 10% formaldehyde for 10 min. The fixed cells were washed with PBS and stained with 2% alizarin red S (pH 4.2) (A5533, Sigma-Aldrich) for 15 min at room temperature. They were then washed with deionized H_2_O, and red-stained cells were photographed under microscope.

To induce BMSCs’ chondrogenesis, cells were cultured in high-density cell aggregates to form a BMSC micromass. The micromass culture was then supplemented with chondrogenic medium (SH30889.02, Thermo) for a duration of 21 days. The accumulated glycosaminoglycan (GAG) levels of BMSCs were measured using Alcian blue (AB) staining. Briefly, the cells were fixed with 10% formaldehyde. The fixed cells were washed with PBS and stained with 0.0018 M H_2_SO_4_ for 30 min. Then, the acid solution was completely removed before adding AB solution (1% AB 8GX in 0.0018 M H_2_SO_4_). The staining was maintained for 3 h. After excess dye was removed, cells were then washed twice in PBS, and the blue-stained cells were photographed under microscope.

For adipogenic differentiation, BMSCs were cultured in Dulbecco’s modified Eagle’s high-glucose medium (Gibco, Paisley, UK) supplemented with 10% FBS, 10^−6^ M dexamethasone, 0.5 mM methyl-isobutyl-methyl-xanthine, 0.2 mM indomethacin, and 10 mg/mL of insulin for 21 days. Oil red O staining was used for staining of the fat content of BMSCs. Briefly, cells were washed twice with PBS and fixed with 10% formaldehyde. Oil red O solution (O1391, Sigma-Aldrich) was added for 10 min. After excess oil red O was removed, cells were washed twice in PBS, and the red-stained cells were photographed under microscope.

### 2.5. Effect of HA Treatment on CD44 Expression and Cell Viability

The HA-coated plate was first prepared by loading 0.5, 1, or 2 mg/mL of HA onto each well of a 12-well tissue culture plate. The HA solution was evenly distributed on the surface of the well and then put into a hood until it completely dried. Later, P2 BMSCs were seeded onto the plate at a density of 1 × 10 ^4^ cells/well. The medium was changed every 3 days until the seventh day of culture. The BMSCs cultured on a non-coated plate were used as controls. The cellularity and morphology of cultured BMSCs were imaged. The expression of the CD44 surface marker following HA treatment was accessed after 7-day culture by flow cytometry, as described above. 

The CD44 protein expression of BMSCs in the control and different HA treatment groups were assessed via immunohistochemical staining. After 7-day culture, the cells of each group were fixed in 4% paraformaldehyde and dehydrated in a gradient ethanol series. Then, immunohistochemical stain was permeabilized in 0.1% (*v*/*v*) Triton X-100 (T8787, Sigma-Aldrich), blocked with 5% (*v*/*v*) donkey serum (AB7475, Abcam), and rinsed in PBS containing 2% (*v*/*v*) BSA. Goat polyclonal antibodies for CD44 were used as a primary antibody at a 1:500 dilution, followed by the diaminobenzidine (DAB) secondary antibody (EnVisionTM System-HRP) to detect protein expression.

For cell viability, at day 7, the viable cell number in the respective groups was counted using thiazolyl blue tetrazolium bromide (MTT; Invitrogen, Carlsbad, CA, USA) following the manufacturer’s instructions. Briefly, MTT reagent was added to each sample and incubated for 3 h to allow the formation of MTT formazan. The resulting formazan was educed with dimethyl sulfoxide (DMSO; Sigma-Aldrich, Inc., St. Louis, MO, USA), and the absorbance of each solution was measured at a wavelength of 595 nm with a microplate reader (Bio-Rad, Hercules, CA, USA) in quadruplicate.

### 2.6. Effect of HA on BMSCs’ Glycosaminoglycan Synthesis 

The accumulated GAG level was measured via Alcian blue staining. After different HA treatments, BMSCs were fixed with 10% formaldehyde for at least 30 min, rinsed with distilled water, and incubated in 0.0018 M H_2_SO_4_ for 30 min. Then, the acid solution was removed completely before adding Alcian blue solution (1% Alcian blue 8GX in 0.0018 M H_2_SO_4_) (Sigma-Aldrich). The staining step took 3 h, followed immediately by washing with 0.018 M H_2_SO_4_ for another 3 h to remove redundant dye. Finally, the bound dye was eluted with dissociation buffer (4 M guanidine hydrochloride in 33% 1-propanol with 0.25% Triton X-100) (Sigma-Aldrich). The absorbance of each sample was then measured at 600 nm using a microplate reader in quadruplicate, and the results in each group were normalized by the cell number obtained from the previous MTT assay.

### 2.7. Effect of HA on BMSCs’ Chondrogenic Gene Expression

P2 BMSCs were cultured on a coated plate containing 0.5, 1, or 2 mg/mL of HA for a total culture period of 7 days. Total RNA of cells after various treatments was extracted by TRIzol^®^ reagent and then stored at –80 °C for later use. RNA (500 ng) was then reverse-transcribed in a 20 μL reaction mixture. Reverse transcription was performed according to the protocol described by the manufacturer (Superscript III Kit, Invitrogen). Aliquots of complementary DNA (cDNA) specimens in each group were further amplified by real-time PCR for quantitative gene expression levels in a qRT-PCR device (Applied Biosystems) SuperScript III platinum SYBR Green One-Step qRT-PCR kit (Life Technologies, CA, USA). Specific primers for type II collagen (*Col2a1*), aggrecan (AGN), and β-actin are shown in Table 1. The relative change in gene expression was determined via the comparative 2^–ΔΔ^*^C^*^T^ method, where ^Δ^*^C^*^T^ = CT, target—CT, β-actin and ^Δ(Δ^*^C^*^T)^ = Δ*C*T, stimulated ^−Δ^*^C^*^T^, control. β-actin was used as the internal control.

### 2.8. Rabbit Chondral Defect Model

**Figure 1:**
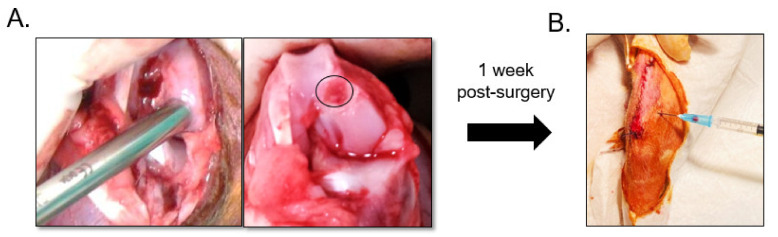
In vivo articular cartilage repair by intraarticular injection of bone-marrow-derived stem/stromal cells (BMSCs) and hyaluronic acid (HA). (**A**) A chondral defect measuring 3 mm in diameter and 1 mm in depth (black circle) was created at the rabbit’s femoral condyle using a trocar. (**B**) Intra-articular injection of BMSCs and HA was performed 1 week post-operatively.

A total of 24 female skeletally mature rabbits (age 14–16 weeks; weight 3–3.5 kg) were used. A prior sample size calculation was performed using G* Power analysis conducted after the preliminary experiment (paired *t*-test; α = 0.05; power = 0.85) revealed an effect size of 1.7 and suggested *N* = 6 animals. All surgeries were performed using a standard surgical routine in the same operating room of the animal surgery center. Surgery was performed under anesthesia with intramuscular (IM) injection of a mixture of 50 mg/kg of ketamine and 10 mg/kg of xylazine. After shaving each animal’s right knee joint, the surgical area was sterilized with iodine-alcohol. Under sterile conditions, a chondral defect measuring 3 mm in diameter and 1 mm in depth was created at the animal’s medial femoral condyle using a cylindrical trocar (Figure 1A). The chondral defect was created in the weight-bearing portion of the femoral medial condyle to mimic articular cartilage damage due to physical overload or stress, and the operation was performed on the right knee joint. During the operation, only the cartilage flap was removed, but the subchondral bone remained intact without penetration. After completion of the procedure, the patella was returned to its normal position, and the joint capsule, subcutaneous tissue, and skin were sutured to close the wound. Post-operatively, penicillin (40,000 IU/kg for 5 days) and ketoprofen (2.2 mg/kg for 3 days) were administered.

### 2.9. In Vivo Intra-Articular Injection of BMSCs and HA 

One-week post-surgery, the 24 rabbits were randomly divided into 4 groups of 6 animals each by injecting different therapeutic substances into the lateral joint cavity using the standard injection protocol (Figure 1B). The four groups were as follows: (1) 0.5 mL of PBS injection (control); (2) 3 × 10^6^ BMSCs suspended in 0.5 mL of PBS; (3) 0.3 mL (0.1 mL/kg) of HA (pH 6.8, 650–1000 kDa, ARTZDispo, Seikagaku, Ibaraki, Japan); (4) 3 × 10^6^ BMSCs suspended in 0.3 mL of HA. Twelve weeks after injection, the rabbits were euthanized and the femur were harvested for macroscopic and histological analysis.

### 2.10. Macroscopic Analysis 

For macroscopic analysis, the knee joints were harvested and digital photographs of the specimens were taken. An International Cartilage Repair Society (ICRS) macroscopic cartilage assessment score was applied to evaluate the repair process of the defect site based on the degree of defect repair, integration to the border zone, the defect margin, and macroscopic appearance (Table 2) [25]. Each specimen was blindly examined by 3 independent researchers, and the scores of each specimen were averaged for comparison. Following macroscopic assessment, each specimen was fixed in 10% buffered neutral formalin, decalcified, and embedded in paraffin for routine histological sectioning.

### 2.11. Histological Analysis

All specimens were fixed in 10% buffered formalin for at least 3 days, decalcified in 5% nitric acid, embedded in paraffin, and sectioned. Histological sections (5 mm) were stained with either H&E or TB. The H&E histological stain was used to evaluate the integrity of the cartilage interface and integration of the repair tissue. Moreover, the cellularity, collagen alignment, cell morphology, and overall organization of the repaired tissue were revealed. On the other hand, cationic TB staining was used to visualize proteoglycan synthesis in the repaired tissue due to its high affinity for sulfate groups in proteoglycans [26]. Thus, metachromatic staining with TB indicates a cartilaginous matrix and the degree of positive staining corresponds with the amount of synthesized proteoglycans [26]. Using the H&E-stained sections, regenerated cartilage was scored based on the ICRS Visual Histological Assessment Scale (Table 3) [27]. Specimens were evaluated independently by 3 individual investigators who were blinded to the treatment assignment and numerical data. The score for each parameter was calculated by averaging the 3 scores.

### 2.12. Statistical Analysis

For the quantitative assay, each data point was derived from three independent experiments or an experiment of quadruplicate assay and was presented as the mean with standard deviation. All analyses were performed using GraphPad Prism 8.0 (San Diego, CA, USA). Statistical significance was set at a *p*-value of <0.05. The gross grading and histological data were analyzed using one-way analysis of variance, followed by the multiple-comparison Scheffé test. The Dunnett test was used when gross grading and histological scores were compared between the control and treatment groups. Groups labeled with asterisk superscript letters indicated that the statistical difference between the two groups had a *p*-value of <0.05, which was considered significantly different.

## 3. Results 

### 3.1. In Vitro

#### 3.1.1. Characterization of BMSC Surface Marker Identification and Differentiation Assay

**Figure 2:**
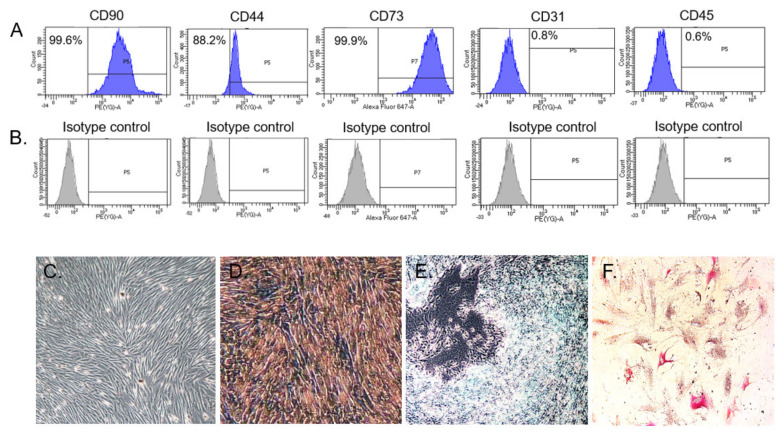
Surface marker expressions and differentiation potential of rabbit bone-marrow-derived stem/stromal cells (BMSCs). (**A**) BMSCs were separately incubated with each surface marker antibody at 4 °C for 30 min and subjected to flow cytometry analysis. Histogram showing the intensity corresponding to the positive staining of each cell surface marker. (**B**) Histogram showing the intensity of the isotype control. The BMSCs displayed a phenotype profile of CD90^+^ CD44^+^ CD73^+^ CD31^−^ CD45^−^. (**C**) Passage 2 adherent BMSCs cultivated in standard culture medium were found to exhibit a fibroblast-like morphology. (**D**) Alizarin red staining, (**E**) Alcian blue staining, and (**F**) Oil red O staining of BMSCs after osteogenic, chondrogenic, and adipogenic induction for 21 days, respectively.

Characterization of harvested BMSCs was performed following the criteria proposed by Dominici et al. [28]. The BMSCs were cultured, and cell morphology was recorded using inverted light microscopy (Leica, Japan). Fluorescence-activated cell sorting surface marker analysis results showed that passage 2 (P2) BMSCs were positive for CD44 (88.2%), CD73 (99.9%), and CD90 (99.6%) and negative for CD31 (0.8%) and CD45 (0.6%) (Figure 2A,B). 

The differentiation potential of the cells toward osteoblasts, chondrocytes, and adipocytes was demonstrated by treating BMSCs with different induction media for a total culture period of 21 days. P2 BMSCs cultivated in standard culture medium served as controls. The cell morphology of P2 BMSCs displayed a well-adherent fibroblast-like phenotype (Figure 2C). In Figure 2D, remarkable reddish precipitation could be observed after alizarin red S (ARS) staining, indicating mineralization deposition of the culture after 21 days of osteogenic induction. In Figure 2E, alcian blue (AB) staining showed abundant glycosaminoglycan (GAG) deposition in the high-density micromass culture after 21 days of chondrogenic induction. Finally, adipogenic-induced cells exhibited red oil droplets after oil red O staining after 21 days of adipogenic induction (Figure 2F). 

#### 3.1.2. Effect of HA on CD44 Expression and Cell Viability 

**Figure 3:**
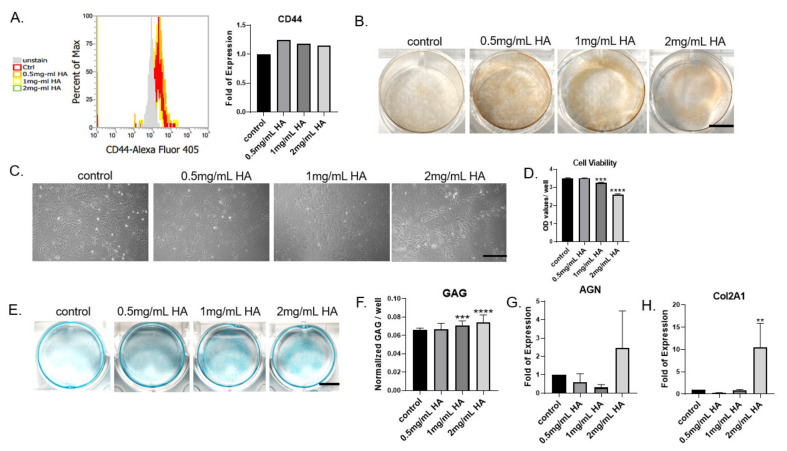
Bone-marrow-derived stem/stromal cells (BMSCs) were exposed to a medium containing different concentrations (0.5–2 mg/mL) of hyaluronic acid (HA) for 7 days, and their effects on BMSCs’ surface marker expression, CD44 protein expression, cellular morphology, cell viability, accumulated glycosaminoglycan (GAG) content, and chondrogenic gene expression levels were analyzed. (**A**) Histograms from flow cytometry analysis showing the percentage of cells positive for the CD44 surface marker increased after HA supplement compared to controls. (**B**) The immunostaining results of CD44 in each group. Cells treated with HA supplement demonstrated increased levels of CD44 expression compared to untreated controls (scale bar 5 mm). (**C**) Microscopic images representing the cellularity and cell morphology of BMSCs at day 7 (scale bar 100 μm). (**D**) At day 7, the cell number significantly decreased in cells treated with high concentrations of HA (1 and 2 mg/mL). (**E**) Alcian blue staining showing glycosaminoglycan (GAG) synthesis of BMSCs of the control and HA supplement groups after 7-day cultivation (scale bar 5 mm). (**F**) Quantitation of normalized GAG synthesis/cell. (**G**,**H**) Quantitative expression profiles of aggrecan (AGN) and collagen II (Col2A1) of cultured BMSCs. Values are presented as the mean ± SD. OD, optical density. ** *p* < 0.01, *** *p* < 0.001, and **** *p* < 0.0001.

Flow cytometry demonstrated a general increase in the level of CD44 was in HA treatment groups compared to controls after culturing for 7 days (Figure 3A). At day 7, the highest level of CD44 (1.25-fold) was observed in BMSCs treated with 0.5 mg/mL of HA compared to controls. No proportional increase in BMSCs’ CD44 expression could be observed when the HA concentration increased from 1 mg/mL (1.18-fold) to 2 mg/mL (1.15-fold). Figure 3B shows the immunohistochemical staining results of BMSCs’ CD44 expression in HA-treated groups. The results were in line with flow cytometry outcomes showing that cells supplemented with 0.5 mg/mL of HA have a higher CD44 expression level than controls. As shown in Figure 3C, higher cellularity was found in BMSCs cultured in control medium compared to HA-treated groups. However, no changes in the cellular morphology of BMSCs were observed between controls and different HA treatment groups. For quantitative analysis, the cell proliferation rate decreased significantly in BMSCs treated with 1 mg/mL (3.26 ± 0.03) and 2 mg/mL (2.61 ± 0.04) of HA compared to controls (3.49 ± 0.05) after 7-day culture (Figure 3D). 

#### 3.1.3. Effect of HA on Cell GAG Expression and Chondrogenic Gene Expression 

To evaluate the effect of HA on promoting chondrogenic gene expression of BMSCs, cells were cultured for up to 7 days in either a control medium or a medium containing 0.5, 1, and 2 mg/mL of HA. The GAG level was normalized to the cell number in each group obtained from thiazolyl blue tetrazolium bromide (MTT) assay. As shown in Figure 3E,F, the accumulated GAG levels increased significantly in BMSCs treated with 1 mg/mL (0.07 ± 0.005) and 2 mg/mL (0.074 ± 0.008) of HA compared to controls (0.066 ± 0.002), showing the stimulatory role of HA in GAG expression. Furthermore, we found the highest level of collagen II (Col2A1) gene expression as a consequence of exposure to 2 mg/mL of HA, with values up to 2.46-fold compared to controls (Figure 3G). Although similar trends were observed in BMSCs cultured with 2 mg/mL of HA supplement, the difference of the aggrecan (AGN) gene expression level among different treatment groups was not significant (Figure 3H). 

### 3.2. In Vivo

#### 3.2.1. Macroscopic Outcome 

**Figure 4:**
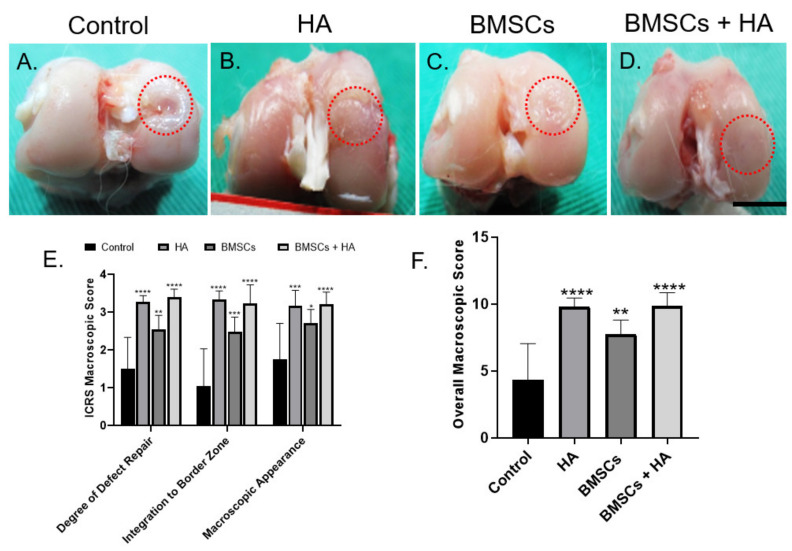
In vivo cartilage repair by the injection of BMSCs and hyaluronic acid supplement. Gross view and grading scale of repaired regions: (**A**–**D**) Macroscopic coronal views of representative defects and appearances after treatment in sham (control), HA, BMSC, and combined BMSC injection 12 weeks post-operatively (scale bar 10 mm). Red circles indicate cartilage defect regions of representative specimens from each group. (**E**,**F**) Quantitative gross grading scale. The bars indicate the mean ± standard deviation (*n* = 6) for each group. HA, hyaluronic acid, BMSCs, bone-marrow-derived stem/stromal cells. * *p* < 0.05, ** *p* < 0.01, *** *p* < 0.001, and **** *p* < 0.0001.

Representative gross views of rabbit knee joints are shown in Figure 4A–D. Twelve weeks after injection, limited repair tissue was noted in the control group treated with phosphate-buffered saline (PBS) injection. Grossly, the articular condyle was coronally concave, the defect margins were clearly distinguishable, and cartilage-like tissue was scarcely detected (Figure 4A). For rabbits treated with isolated HA or BMSC injection, cartilage regeneration was better than in controls. In both groups, the defects were filled with varying degrees of reparative tissue (Figure 4B,C). Some fissures and depressed areas were still observed at the periphery of the defects, indicating incomplete tissue healing. In contrast, in the combined BMSC and HA group, the defect was mostly filled with new cartilage-like tissue. The reparative tissue was connected well with adjacent unoffended cartilage tissue. Moreover, the condylar articular surface was relatively smooth and intact, resembling the normal articular surface (Figure 4D). Quantitatively, the macroscopic score for the isolated HA (9.78 ± 2.72), BMSC (7.74 ± 1.05), and combined HA and BMSC (9.86 ± 0.98) treatment groups was significantly higher than that for untreated controls (4.3 ± 2.72) (Figure 4E). Despite a higher gross score being achieved in the combined BMSC and HA group, no significant difference existed between these three treatment groups (Figure 4F). 

#### 3.2.2. Histological Evaluation of Defect Healing 

**Figure 5:**
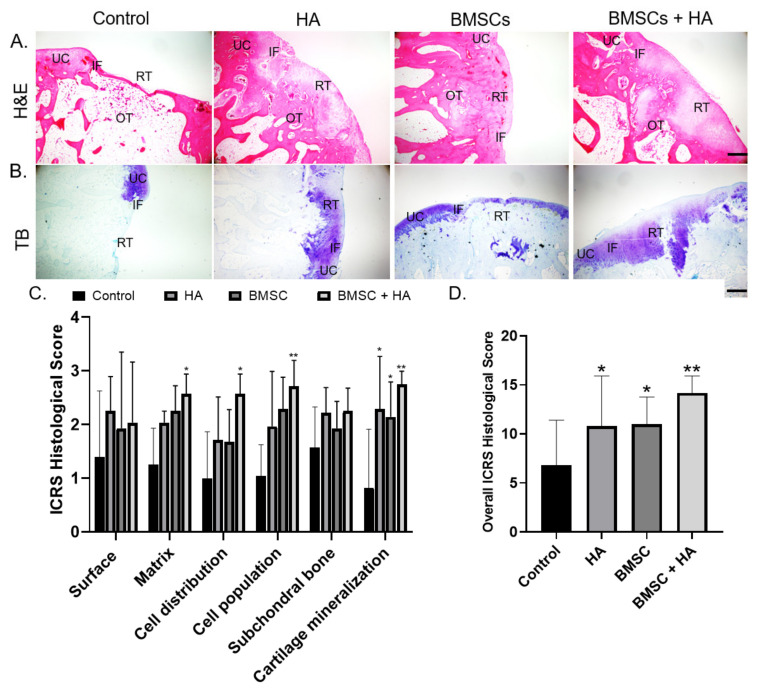
(**A**) Hematoxylin and eosin (H&E) staining of histological sections of representative defects from the control group and the three treatment groups treated with hyaluronic acid (HA), bone-marrow-derived stem/stromal cells (BMSCs), and combined BMSCs and HA. In untreated controls, the defect was obvious without notable regenerated tissue. In the treatment groups, more repair tissue was found filling the defects. Scale bars: 100 μm. (**B**) Toluidine blue staining of histological sections of representative defects of the three groups. Scale bars: 100 μm. UC, unoffended cartilage; IF, interface; RT, repaired tissue; and OT, subchondral bone. (**C**,**D**) International Cartilage Repair Society scores evaluating repair tissue in the control, HA, BMSC, and combined BMSC and HA groups at 12 weeks. The bars indicate the mean ± standard deviation (*n* = 6) for each group. * *p* < 0.05 and ** *p* < 0.01.

The histological images of each group were analyzed using hematoxylin and eosin (H&E) and toluidine blue (TB) stains, specifically classified into four distinct zones: unoffended cartilage (UC), cartilage interface (IF), repair area (RT), and osteochondral interface (OT). Figure 5A,B shows the representative photomicrographs of H&E (upper panel) and TB (lower panel) staining of each group. In the control group, the articular condyle was concave with a discontinuous surface over the defect site, showing no sign of cartilage regeneration. In contrast, significantly more regenerated cartilaginous tissues was found filling the defects in the treatment groups (HA, BMSCs, and combined BMSCs and HA). Moreover, the repair tissue was adequately integrated with the adjacent native cartilage and subchondral bone. Figure 5B shows repaired regions stained with TB. Limited repair tissue could be noted in the controls, which was negatively stained for TB. These results were consistent with H&E observations, indicating superior healing results in the treatment groups compared to controls. Interestingly, there was a difference in TB metachromasia among treatment groups. As shown in Figure 5B, compared to isolated HA and combined BMSC and HA groups, repaired regions in the isolated BMSC group consisted primarily of fibrous tissue that was negatively stained for TB. Quantitative analysis with the International Cartilage Repair Society (ICRS) histological scoring revealed that histological evaluation of the repaired tissue was the best in the combined BMSC and HA group (14.75 ± 1.8), followed by isolated HA (12.28 ± 3.5) and BMSC (11.5 ± 3.4) groups (Figure 5C,D). 

## 4. Discussion 

This current study reveals the efficacy of intra-articular (IA) administration of BMSCs, HA, or their combination for treating chondral defects in a rabbit model. This study is distinct from previous reports because it delineates the essentiality of BMSCs, HA, or their combination for cartilage healing. The results revealed that BMSCs suspended in HA show superior tissue healing outcomes than isolated BMSC or HA injection in terms of gross grading and histological outcomes. The data also support HA’s role as an ideal chondrogenic adjuvant in facilitating cartilaginous healing, not only relying on HA’s cytoprotective effects on chondrocytes but also relying on its positive impact on BMSCs’ chondrogenic differentiation. 

Articular cartilage injury is a common clinical problem that may cause pain and functional disability of affected joints. Since cartilage is an avascular tissue, its self-healing capacity is limited. As a result, chondral lesions are likely to deteriorate and progress to osteoarthritis. Numerous efforts have been made to improve the inherently limited healing capacity of articular cartilage by using stem cells of different origins (mesenchymal, synovial, adipose) [6,13,18,20,29,30,31,32]. Unlike conventional treatment modalities, which need open surgery for cells or scaffold transplantation, direct stem cell injection emerges as a simple, minimally invasive, and attractive therapeutic approach for treating chondral defects in recent years. Moreover, many studies have reported promising results of stem-cell-based injection therapy in promoting cartilage repair and regeneration [12,13,17,18,20,29,30,31,33]. Mcllwraith et al. showed that full-thickness chondral defects in an equine model treated with microfracture followed by IA BMSC injection have superior outcomes than microfracture alone [32]. Nam et al. reported similar findings showing that IA injection of BMSCs could augment the effects of bone marrow stimulation surgery in their caprine model [34]. Despite growing evidence demonstrating the positive influence of injection-based stem cell therapy on the regeneration of damaged cartilaginous tissue, discrepancies with the literature are found regarding the efficacy and longevity of the injected stem cells after in vivo administration. The duration varies widely from 7 days to 12 weeks, as previously reported [27,28,32,33]. Some authors postulated that the discrepancies that existed among different studies may be attributed to different experimental settings, including animal model, cell type, cell number, technique for labeling and detection, timing of injection, and the presence of an adjuvant for cell delivery [33]. 

In the current study, we found that the combinatory use of BMSCs and HA improves the cartilaginous healing of chondral defects, both macroscopically and microscopically. The results were in line with other stem-cell-based injection studies with rat, rabbit, and canine models [17,18,19]. Interestingly, most studies consistently reported the superior efficacy of combined BMSCs and HA on chondral defect healing than that of isolated stem cells or HA alone. In 2015, Yamasaki et al. reported that bone marrow stimulation surgery followed by direct injection of BMSCs in hyaluronic acid resulted in an improvement of the quality of cartilage repair (hyaline cartilage) when compared to HA alone (fibrocartilage) in a canine animal model [19]. Li et al. injected allogenic BMSCs intra-articularly to treat femoral chondral defects in a canine model, and the results favored the combined use of HA on cartilage healing compared to HA alone [18]. Collectively, these studies have already pointed out the importance of the combinatory use of a chondrogenic adjuvant that would lead to significant better healing outcomes than HA alone. However, when looked at in depth, we found that limited studies have explored and compared the chondrogenic adjuvant role of HA in augmenting the therapeutic efficacy of BMSCs, as what we did in the present study. In our current study, we directly compared the therapeutic efficacy of BMSCs with and without HA supplement, which is absent in other previous reports. For future clinical application, a direct comparison of the therapeutic efficacy of BMSCs alone or BMSCs supplemented with a chondrogenic adjuvant would be necessary in order to have a better understanding concerning the individual role of BMSCs and chondrogenic adjuvants in stem-cell-based treatment regimens.

HA is a large glycosaminoglycan (GAG), which acts as one of the crucial components of the cartilage extracellular matrix [35]. Clinically, HA has long been used in patients with osteoarthritis to reduce pain and joint stiffness. Besides acting as a lubricant, HA supplementation helps to restore the biological environment of the joint by reducing friction and improving viscoelasticity [36,37]. Additionally, HA may improve cartilage healing by coating the cartilage surface and localizing in the cartilage extracellular matrix among the collagen fibrils and proteoglycan [38,39]. From this standpoint, HA undoubtedly becomes an ideal suspension solution to serve as a biological carrier for stem cell delivery. Moreover, HA also displays significant positive effects on chondrocytes’ viability and metabolic activities [22]. Akmal et al. showed that low concentrations of HA (0.1–1 mg/mL) increase chondrocytes’ DNA, sulfated GAG, and hydroxyproline synthesis [22]. Liu et al. reported HA’s protective effects on chondrocytes against death induced by bupivacaine at supraphysiologic temperatures [40]. The chondroprotective and cytoprotective effects of HA were further supported by Grishko et al., who showed that HA pre-treatment can promote chondrocytes’ viability by decreasing mitochondrial DNA damage and preserving ATP levels [41]. 

To further improve the clinical applicability of stem-cell-based injection therapy, it is of paramount importance to improve stem cell targeting and survival after in vivo administration. Following IA administration, the ability of the injected stem cells for homing toward the site of injury or inflammation relies upon their cellular interaction with the chemical components of the microenvironment. In fact, both chondrocytes and BMSCs express CD44 on their cell surface, a major HA receptor that is involved in cell–cell and cell–matrix interactions [23,42]. According to Sackstein et al., the CD44–HA interaction is essential for directing the migration of exogenous BMSCs to damaged tissues, and any deficit in receptor binding to selectins and/or their ligands (L-selectin, and their E-selectin ligand CD44) may make BMSCs nonfunctional [43]. Corradetti et al. proposed the pivotal role of HA as a mediator for guiding BMSCs towards the inflammatory site. In their experimental setting, pre-culture of BMSCs on a HA-coated surface successfully led to overexpression of the HA receptor on the BMSCs’ membrane. In this study, we demonstrated the positive effect of HA in upregulating BMSCs’ CD44 surface marker expression, both on flow cytometry analysis and on immunohistochemical staining (Figure 3A,B). The results support our hypothesis that HA-treated BMSCs would demonstrate significantly higher inflammatory targeting abilities, both in vitro and in vivo. On the basis of these studies, it is reasonably to believe that BMSCs, when injected with HA in vivo, are expected to show adequate homing ability, which would be beneficial for subsequent chondral healing. 

There are a few limitations of this study. First, despite significant better tissue healing found in the combined BMSC and HA group, the regeneration of chondral defects did not achieve a completely normal articular cartilage. Perhaps more than a single time point is needed to evaluate the sequential tissue-healing process in different treatment groups. Second, in this study, we did not pre-culture BMSCs with HA to induce CD44 overexpression before injection. As reported by Marquass et al., injection of chondrogenic-induced BMSCs may lead to better cartilage healing outcomes than ordinary BMSCs [44]. In the future, further experiments would be necessary to compare the therapeutic efficacy of BMSCs with or without HA pre-treatment. Finally, the mechanical properties of the repaired cartilage were not measured. However, compression testing techniques would be difficult to apply because of the curvature in the cartilage surface and the irregular shape of the repaired cartilage. 

## 5. Conclusions

In the current study, the results of in vitro tests revealed the effects of HA–BMSC interaction, particularly on CD44 expression and chondrogenic phenotypic expression. We believe that the positive effects of HA in promoting the chondrogenic differentiation of BMSCs as well as CD44 expression would be a potential niche to be exploited to improve the therapeutic efficacy of stem-cell-based therapies. For the in vivo experiment, the results confirmed the feasibility of effective delivery of BMSCs in an HA suspension to achieve cartilage defect healing in a rabbit model. Furthermore, the individual role of cells and HA in cartilage healing could be clearly delineated through direct comparison between experimental groups. On the basis of these results, a novel therapeutic approach to potentiate the application of combined HA and BMSC therapy is expected to expedite the clinical translation of cartilage repair.

## Figures and Tables

**Table 1:** Primers for rabbit chondrogenic marker messenger RNA (mRNA) detection.

Gene	Primer Sequence	Size (Base Pair)
*Col2a1*	F:GCACCCATGGACATTGGAGGG R:GACACGGAGTAGCACCATCG	366
*AGN*	F:GAGGAGATGGAGGGTGAGGTCTTT R:CTTCGCCTGTGTAGCAGCTG	313
*β-ACTIN*	F:CAACTGGGACGACATGGAGAAG R:TGAACGTCTCGAACATGATCTG	152

**Table 2:** International Cartilage Repair Society macroscopic evaluation of cartilage repair.

Features	Grade
Degree of defect repair	
In level with surrounding cartilage	4
75% repair of defect depth	3
50% repair of defect depth	2
25% repair of defect depth	1
0% repair of defect depth	0
Integration to border zone	
Complete integration with surrounding cartilage	4
Demarcating border < 1 mm	3
3/4th of graft integrated, 1/4th with a notable border > 1 mm width	2
1/2 of graft integrated with surrounding cartilage, 1/2 with a notable border > 1 mm	1
From no contact to 1/4th of graft integrated with surrounding cartilage	0
Macroscopic appearance	
Intact smooth surface	4
Fibrillated surface	3
Small, scattered fissures or cracks	2
Several small or few but large fissures	1
Total degeneration of grafted area	0
Overall repair assessment	
Grade I: normal	12
Grade II: nearly normal	11–8
Grade III: abnormal	7–4
Grade IV: severely abnormal	3–1

**Table 3:** International Cartilage Repair Society Histological Assessment Scale.

Feature		Score
I. Surface		
	Smooth/continuous	3
	Discontinuities/irregularities	0
II. Matrix		
	Hyaline	3
	Mixture: hyaline/fibrocartilage	2
	Fibrocartilage	1
	Fibrous tissue	0
III. Cell distribution		
	Columnar	3
	Mixed: columnar/cluster	2
	Cluster	1
	Individual cells/disorganized	0
IV. Cell population		
	Predominantly viable	3
	Partially viable	1
	<10% viable	0
V. Subchondral bone		
	Normal	3
	Increased remodeling	2
	Bone necrosis/granulation tissue	1
	Detached/fracture/cells at base	0
VI. Cartilage mineralization (calcified cartilage)		
	Normal	3
	Abnormal/inappropriate	0

## Data Availability

Data is contained within the article.
